# Supplementary material for: Infection Prevention and Control in Three Tertiary Healthcare Facilities in Freetown, Sierra Leone during the COVID-19 Pandemic: More Needs to Be Done!
Source: Int J Environ Res Public Health. 2022 Apr 26;19(9):5275. doi: 10.3390/ijerph19095275 (PMC9105082; doi:10.3390/ijerph19095275)
Supplement: Supplementary file 1 [file ijerph-19-05275-s001.zip › ijerph-1624945-supplementary.pdf]

Supplementary Table S1: Baseline level of compliance for each core component of the IPC programs at the three tertiary health facilities in Freetown, Sierra Leone, 2021.

**Core Components**	Score Interpretations		
	**Connaught**	**ODCH**	**PCMH**
**IPC program**	**Intermediate**	**Intermediate**	**Intermediate**
IPC focal, team and committee	50	55	50
Dedicated budget	0	0	0
Access to microbiology laboratory	5	5	5
Component total scores (N=100)	55	60	55
**IPC guideline**	**Basic**	**Basic**	**Basic**
Availability of guidelines	17.5	17.5	17.5
Guideline consistent with international standards	10	10	10
Stakeholders' involvement in the development	7.5	7.5	7.5
Training of HCW on guidelines	10	10	10
Component total scores (N=100)	45	45	45
**IPC education and training**	**Basic**	**Basic**	**Basic**
Personnel to lead training	20	20	10
Frequency of training	15	10	10
Methods of training	10	10	10
Component total scores (N=100)	45	40	30
**HAI surveillance**	**Inadequate**	**Inadequate**	**Inadequate**
Surveillance a component of IPC program	5	5	10
Prioritization activities	0	0	5
Mode of Surveillance	2.5	2.5	0
Component total scores (N=100)	7.5	7.5	15
**Multimodal strategy**	**Basic**	**Basic**	**Basic**
Utilization of multimodal strategy	15	15	15
Elements of the multimodal strategy	20	20	20
Strategies included bundle and checklist	10	10	10
Component total scores (N=100)	45	45	45
**Monitoring/audit of IPC practices**	**Basic**	**Basic**	**Basic**
Monitoring plans with goals and targets	10	10	10
Hand Hygiene compliance	5	5	5
Cleaning of ward environment	5	5	5
Disinfection and Sterilization	5	5	5
Waste management	5	5	5
Hand hygiene self-assessment framework	2.5	2.5	2.5
Feedback on monitoring activities	5	7.5	5
Component total scores (N=100)	37.5	40	37.5
**Workload, staffing and bed occupancy**	**Basic**	**Basic**	**Basic**
Staffing	0	0	0
Bed occupancy	40	40	30
Component total scores (N=100)	40	40	30
**Built environment and supplies**	**Intermediate**	**Basic**	**Basic**
Water	7.5	0	0
Hand hygiene and sanitation facilities	15	10	2.5
Power supply, ventilation and cleaning	12.5	15	12.5

Patient placement and PPE	5	5	5
Medical waste management and sewage	11	11	11
Decontamination and sterilization	7.5	5	7.5
Component total scores (N=100)	58.5	46	38.5
**Facility overall Scores**	**Basic**	**Basic**	**Basic**

Maximum score for each component was 100. Core component score interpretations: 0-25% Inadequate; 25.1-50% Basic; 50.1-75% Intermediate; and 75.1-100% Advanced. IPC = infection prevention control; HAI = healthcare associated infections.
